# P2Y_6_ Receptors Regulate CXCL10 Expression and Secretion in Mouse Intestinal Epithelial Cells

**DOI:** 10.3389/fphar.2018.00149

**Published:** 2018-02-28

**Authors:** Mabrouka Salem, Alain Tremblay, Julie Pelletier, Bernard Robaye, Jean Sévigny

**Affiliations:** ^1^Département de Microbiologie-Infectiologie et d’Immunologie, Faculté de Médecine, Université Laval, Québec City, QC, Canada; ^2^Centre de Recherche du CHU de Québec – Université Laval, Québec City, QC, Canada; ^3^Institut de Recherche Interdisciplinaire en Biologie Humaine et Moléculaire, Université Libre de Bruxelles, Gosselies, Belgium

**Keywords:** intestinal epithelial cells (IEC), P2Y_6_, CXCL10, TLR, nucleotides

## Abstract

In this study, we investigated the role of extracellular nucleotides in chemokine (KC, MIP-2, MCP-1, and CXCL10) expression and secretion by murine primary intestinal epithelial cells (IECs) with a focus on P2Y_6_ receptors. qRT-PCR experiments showed that P2Y_6_ was the dominant nucleotide receptor expressed in mouse IEC. In addition, the P2Y_6_ ligand UDP induced expression and secretion of CXCL10. For the other studies, we took advantage of mice deficient in P2Y_6_ (*P2ry6*^-/-^). Similar expression levels of P2Y_1_, P2Y_2_, P2X2, P2X4, and A_2A_ were detected in *P2ry6*^-/-^ and WT IEC. Agonists of TLR3 (poly(I:C)), TLR4 (LPS), P2Y_1_, and P2Y_2_ increased the expression and secretion of CXCL10 more prominently in *P2ry6*^-/-^ IEC than in WT IEC. CXCL10 expression and secretion induced by poly(I:C) in both *P2ry6*^-/-^ and WT IEC were inhibited by general P2 antagonists (suramin and Reactive-Blue-2), by apyrase, and by specific antagonists of P2Y_1_, P2Y_2_, P2Y_6_ (only in WT), and P2X4. Neither adenosine nor an A_2A_ antagonist had an effect on CXCL10 expression and secretion. Macrophage chemotaxis was induced by the supernatant of poly(I:C)-treated IEC which was consistent with the level of CXCL10 secreted. Finally, the non-nucleotide agonist FGF2 induced MMP9 mRNA expression also at a higher level in *P2ry6*^-/-^ IEC than in WT IEC. In conclusion, extracellular nucleotides regulate CXCL10 expression and secretion by IEC. In the absence of P2Y_6_, these effects are modulated by other P2 receptors also present on IEC. These data suggest that the presence of P2Y_6_ regulates chemokine secretion and may also regulate IEC homeostasis.

## Introduction

Dysregulation of immune response is an important contributor to inflammatory bowel diseases in which the epithelium plays central functions. The intestine is bordered by intestinal epithelial cells (IECs) that represent the first line of defense against environmental pathogens. In addition to acting as a physical barrier to prevent passage of luminal contents, IECs are crucial for maintaining intestinal homeostasis. For example, IECs express a wide range of pattern-recognition receptors (PRRs), including Toll-like receptors (TLRs) that are activated by pathogen-associated molecular patterns (PAMPs) (Qureshi and Medzhitov, 2003; Medzhitov, 2007). The activation of these receptors results in the activation of innate immune responses, inducing the expression of pro-inflammatory cytokines, chemokines, and antimicrobial peptides (Fritz et al., 2006; Artis, 2008).

Once injured, or upon mechanical stimulation, epithelial cells such as bronchial and IECs release danger signals such as nucleotides which include adenosine triphosphate (ATP), uridine triphosphate (UTP), and their derivatives (Hazama et al., 1999; Braunstein et al., 2001; Vitiello et al., 2012). These molecules can alert the immune system by initiating tissue inflammation via the activation of plasma membrane P2 receptors (Lazarowski et al., 2003; Kukulski et al., 2011). Based on their structure and distinct signal-transduction mechanism, P2 receptors are divided into P2X (P2X1-7) and P2Y (P2Y_1,2,4,6,11,12,13,14_) subfamilies (Abbracchio et al., 2006). Among the P2YR receptor subtypes, P2Y_6_ is known as a pro-inflammatory receptor which is specifically activated by UDP in human and by UTP and UDP in mouse (Kauffenstein et al., 2010). Several reports suggested an important role of P2Y_6_ receptors in modulating cellular responses to inflammation. P2Y_6_ participates in the regulation of TLR1/2-induced IL-8 secretion from monocytes and, as a consequence, in neutrophil migration (Ben Yebdri et al., 2009). In microglia and astrocytes, P2Y_6_ activation results in the regulation of CCL2 expression (Kim et al., 2011). Moreover, its activation results in the amplification of chemokine secretion induced by lipopolysaccharide (LPS) in the monocytic cell line (THP-1) and in human and mouse monocytes/macrophages (Warny et al., 2001; Kukulski et al., 2007; Bar et al., 2008).

In the context of the gastrointestinal tract, P2Y_6_ receptors were reported to be strongly expressed by colonic epithelial cells in biopsies from patients with inflammatory bowel disease (Grbic et al., 2008). It was also shown that colonic epithelial cell lines challenged by TNF stimulation, to mimic an inflammatory stress, release UDP which activates P2Y_6_ receptor and mediates production of CXCL8 (Grbic et al., 2008). There are also some reports that show increase of chemokine expression in IEC lines upon bacterial infection or stimulation with TNF-α or IL-1α (Jung et al., 1995; Yang et al., 1997) in which it is not excluded that P2Y_6_ may participate.

Other studies suggest that CXCL10 has a strong association with inflammatory diseases and particularly with intestinal inflammation (Laragione et al., 2011; Bondar et al., 2014). In humans, CXCL10 is constitutively expressed by colonic epithelial cells (Dwinell et al., 2001) and it is permanently over-expressed in patients with ulcerative colitis (UC) and Crohn’s disease (CD) (Uguccioni et al., 1999; Ostvik et al., 2013; Singh et al., 2016). CXCL10 is a chemoattractant for activated T cells and monocytes (Suzuki et al., 2007; Zhao et al., 2017). It was shown that CXCL10, by binding to CXCR3 receptors, is responsible for Th1 cell differentiation and trafficking into both, the epithelium and the lamina propria of inflamed colons (Uguccioni et al., 1999; Suzuki et al., 2007). Additionally, CXCL10 is responsible for macrophage chemotaxis in murine models of inflammation: non-alcoholic steatohepatitis and puromycin aminonucleoside nephrosis (Petrovic-Djergovic et al., 2015; Tomita et al., 2016).

Giving the fact that the expression of P2Y_6_ receptor by IEC is increased in inflammatory condition and in biopsies of patients with intestinal inflammation and that it contributes to chemokine release such as CXCL8, we hypothesized that P2Y_6_ receptor plays a role in the secretion of chemokines by IEC and in immune cell migration to the epithelium. We addressed this hypothesis here with primary IEC cultures from mice deficient in the expression of P2Y_6_ receptor. We observed that extracellular nucleotides, via the activation of P2Y_6_ and also of other P2 receptors, regulate the expression and secretion of CXCL10. In addition, our results also show that IEC deficient for P2Y_6_ respond more vigorously to stimulation with several ligands activating other nucleotide receptors as well as a non-nucleotide receptor. In other words, these data suggest that P2Y_6_ activation not only activates the synthesis and release of a chemokine but that the presence of P2Y_6_ also maintains a proper state of activation of these cells, preventing them from overstimulation by several stimuli.

## Materials and Methods

### Reagents and Chemicals

Suramin and Reactive-Blue-2 (RB-2) were obtained from MP Biomedical (Santa Ana, CA, United States). DMEM/F12, advanced DMEM/F12, HEPES, L-glutamine, penicillin, streptomycin, FBS, Dulbecco’s PBS, apyrase, formyl-methionine-leucyl-phenylalanine (FMLP), LPSs, adenosine 5’-triphosphate (ATP), adenosine 5’-diphosphate (ADP), uridine 5’-triphosphate (UTP), uridine 5’-diphosphate (UDP), adenosine, Zm 241385, and fibroblast growth factor 2 (FGF2) were purchased from Sigma–Aldrich (Oakville, ON, Canada). Collagenase type I, SuperScript III, gentamicin, B-27 and N-2 supplements, polyinosinic–polycytidylic acid [poly(I:C)], EDTA, and TRIzol were obtained from Invitrogen (Carlsbad, CA, United States). Collagen type I was purchased from BD Bioscience (San Jose, CA, United States). Y-27632, mrEGF, Wnt-3a, and R-spondin were purchased from R&D Systems (Minneapolis, MN, United States). Noggin and M-CSF were purchased from PeproTech (QC, Canada). SYBR Green and DNAseI were from Roche Diagnostics (Indianapolis, IN, United States). Flagellin was obtained from InvivoGen (San Diego, CA, United States). Oligo(dt)18 was obtained from Fisher Scientific (Ottawa, ON, Canada). MRS 2500, MRS 2179, MRS 2578, AR-C 118925XX, PSB 1114, and 5-BDBD were purchased from Tocris Bioscience (Minneapolis, MN, United States).

### Animals

All experiments were conducted according to the Canadian Council on Animal Care and the protocols were approved by the Animal Care Committee of Laval University. Adult male C57Bl/6 mice 8–12 weeks (Charles River, Pointe-Claire, QC, Canada) were used as control. *P2ry6*^-/-^ mice were bred at our animal facility in CHUL. Previously to the experiments presented in this paper, P2Y_6_-deficient mice obtained from B. Robaye (Université Libre de Bruxelles, Belgium) (Bar et al., 2008) were backcrossed 10 times with C57Bl/6 mice from Charles River. A few backcrosses with WT females were performed to ascertain that the mitochondrial DNA is the same in mutant and control mice. Animals were maintained in a specific pathogen-free environment in a temperature-controlled room (21°C) on a 12-h/12-h light and dark cycle and given unrestricted access to standard diet and tap water. Mice were allowed to acclimate to these conditions for at least 7 days before experimentation.

### Intestinal Epithelial Cell Isolation

Primary IECs were isolated from WT and *P2ry6*^-/-^ mice according to Moon et al. (2014) and Graves et al. (2014) with minor modifications as detailed below. Briefly, the longitudinal muscle layer was removed and the colonic tissue was washed with ice-cold Mg^2+^ and Ca^2+^ free salt solution (PBS). Obtained tissue was incubated with 75 U ml^-1^ collagenase type V for 30 min and the reaction was stopped with Dulbecco’s modified Eagles medium (DMEM/F12) containing 10% v/v fetal bovine serum (FBS), L-glutamine, HEPES, N-2 supplement, B-27 supplement, and antibiotics as before. The digestion mixture was filtered through a 70-μm mesh and the effluent containing crypts was centrifuged twice at 50 × *g* for 5 min at 4°C. The remaining pellet comprising isolated intestinal crypts was suspended in complete growth media DMEM/F12 advance containing 50 ng/ml mrEGF, 1 μg/ml R-spondin, 100 ng/ml noggin, and 5 ng/ml Wnt-3a in the presence of 10 μM Y-27632 as an anoikis inhibitor. The suspended crypts were then plated in a 24-well plate coated with type I collagen at a density of 1000 crypts/well. Cells were incubated at 37°C in a 5% CO_2_ incubator. The media was replaced 48 h later without Y-27632 and the epithelial cells from the crypts were allowed to grow to confluence for 2 days to obtain a monolayer of differentiated IEC. The cells were either collected for qPCR or stimulated as detailed below.

### IEC Stimulation

Intestinal epithelial cells were stimulated for 5 (for qPCR experiments) or 24 h (for ELISA experiments) with the ultrapure TLR agonists poly(I:C) [TLR3] (10 μg/ml), LPS [TLR4] (100 ng/ml), flagellin [TLR5] (100 ng/ml), or with the nucleotide ATP, ADP, UTP, UDP, or adenosine all at the concentration of 100 μM. In some experiments, IEC culture was stimulated with poly(I:C) in the presence of general P2 receptor blockers added 20 min before stimulation, suramin (100 μM), RB-2 (100 μM), apyrase (2 U/ml), or in the presence of specific antagonist of P2Y_1_, P2Y_2_, P2Y_6_, P2X4, or A_2A_ receptor (as described in the legend of the figures).

### Quantitative Real-Time PCR (qRT-PCR) and ELISA

RNA extraction, cDNA synthesis, and quantification were performed as described previously with some modifications (Bahrami et al., 2014). Briefly, total RNA from stimulated or unstimulated IEC monolayer was extracted with TRIzol then quantified with a Quant-iT RNA BR Assay Kit and Qubit Fluorometer. The cDNA was synthesized with SuperScript III from 1 μg of total RNA with oligo (dT)18 as the primer, according to the instructions of the manufacturer (Invitrogen, Carlsbad, CA, United States). Primers specific for the differentiation marker villin, for the ectonucleotidases, and for P2X, P2Y, and P1 receptors were either designed by us and synthetized by Invitrogen (Carlsbad, CA, United States) or purchased from Qiagen (Toronto, ON, Canada), as detailed in **Table 1**. SYBR Green Supermix was used for qRT-PCR. For the negative controls, water was used as template. Standard curves were used to determine mRNA transcript copy number in individual reactions. GAPDH or actin was used to normalize RNA quantities between samples.

**Table 1:** qRT-PCR primers.

Gene	Forward primer	Reverse primer	Amplicon (bp)
*Vil1*	Qiagen	Qiagen	135
*Alpi*	Qiagen	Qiagen	113
*Gapdh*	AACTTTGGCATTGTGGAAGG	ACA CAT TGG GGG TAG GAA CA	223
*Actb*	AGCCATGTACGTAGCCATCC	CTC TCA GCT GTG GTG GTG AA	228
*Entpdl*	AGC TGC CCC TTA TGG AAG AT	TCA GTC CCA CAG CAA TCA AA	123
*Entpd2*	TTC CTG GGA TGT CAG GTC TC	GTC TCT GGT GCT TGC CTT TC	132
*Entpd3*	ACC TGT CCC GTG CTT AAA TG	AGA CAG AGT GAA GCC CCT GA	183
*Entpdl7*	Qiagen	Qiagen	91
*Entpd8*	CCC TTA TGA ACC CCT GAC CT	AAT CCA ACC ACA GGC TCT TG	292
*NtT5e/CD73*	CAG GAA ATC CAC CTT CCA AA	AAC CTT CAG GTA GCC CAG GT	128
*P2ry1*	TCG-TGT-CTC-CAT-TCT-GCT-TG	CGA CAG GGT TTA TGC CAC TT	218
*P2ry2*	TGA CGA CTC AAG ACG GAC AG	GTC CCC TAC AGC TCC CCT AC	108
*P2ry4*	AGACGGGCCTGATGTGTATC	AGG TTC ACA TGC CCT GTA CC	126
*P2ry6*	GGT-AGC-GCT-GGA-AGC-TAA-TG	TTT CAA GCG ACT GCT GCT AA	308
*P2ry12*	GGC-AGC-CTT-GAG-TGT-TCT-TC	ATA ACG TGC TAC CCG ACC TG	130
*P2ry13*	ATA-GAG-AAC-CGG-GAA-CAG-CA	CAA AAC AAA GCT GAT GCT CG	115
*P2ry14*	TTT TGT CGT CTG CTT TGT GC	GCA GCC GAG AGT AGC AGA GT	135
*P2rx1*	CAA CTG TGT GCC CTT CAA TG	GGT ACC ATT CAC CTC CTC CA	114
*Pr2x2*	GCT GGG CTT CAT TGT AGA GC	CCT GTC CAT GCA CAA TAA CG	281
*P2rx3*	ATT TCC TCA AAG GGG CTG AT	GTT CTG CAG CCC AAG GAT AA	204
*P2rx4*	CAC AAC GTG TCT CCT GGC TA	GCC TTT CCA AAC ACG ATG AT	125
*P2rx5*	CTG TCA CTT CAG CTC CAC CA	TTT GTT GTC CAG ACG GTT GA	196
*P2rx6*	TCA CCC GCT AAC CCT GTT AC	TAG TCC CGC TGA AGC TTT GT	242
*P2rx7*	AAT CGG TGT GTT TCC TTT GG	CCG GGT GAC TTT GTT TGT CT	165
*Adora 1*	GTG ATT TGG GCT GTG AAG GT	AGT AGG TCT GTG GCC CAA TG	142
*Adora2a*	TCA ACA GCA ACC TGC AGA AC	GGC TGA AGA TGG AAC TCT GC	186
*Adora2b*	TCT GGC CTT TTG GAG AAG AA	TTT CCG GAA TCA ATT CAA GC	246
*Adora3*	TGT GGA GGG AGT CTC GTC TT	TCC TTC TGT TCC CCA CAT TC	97

Supernatants from IEC stimulated for 24 h were centrifuged (1000 × *g*, 10 min, 4°C) to discard the detached cells. The supernatants were collected and frozen at -80°C until determination of cytokine concentrations by ELISA Kits (R&D Systems, Minneapolis, MN, United States), following the manufacturers’ instructions.

### Isolation and Preparation of Murine Bone Marrow Macrophages

Murine macrophages were isolated as described before (Cho et al., 2012) with some modifications. Briefly, bone marrow-derived monocytes were isolated from tibia and femur harvested from mice. Cells were flushed out with PBS containing 1% FBS then filtered through a 70-μm cell strainer. The single cell suspension was centrifuged 10 min at 500 × *g* then resuspended in macrophage complete media [DMEM/F12 medium supplemented with 10% (v/v) FBS, 100 U/ml penicillin, 100 mg/ml streptomycin, 10 mM L-glutamine, and 10 ng/ml M-CSF]. Cells were seeded on 24-well plates (10^6^ cells per well) and incubated at 37°C in a 5% CO_2_ atmosphere. Four days after seeding the cells, an extra 5 ml of fresh macrophage complete medium was added per plate and incubated for an additional 3 days to get adherent cells constituted of approximately 95% macrophages. To obtain bone marrow-derived macrophages (BMDM), the cell supernatant was discarded and the attached cells were washed with 10 ml of sterile PBS. Then, cell stripper non-enzymatic cell dissociation solution (D-PBS containing 1 mM EDTA) was added to each dish and incubated 5 min at 37°C. This solution contains the divalent cation chelator EDTA that gently dislodges adherent cells as an alternative to trypsin. After detachment of macrophages from the plate, an equal volume of cold DMEM/F12-10 medium was added to the wells then cells were centrifuged at 400 × *g* for 10 min at 4°C. The cells were counted then used for chemotaxis assay.

### *In Vitro* Chemotaxis Assay

Macrophage chemotaxis was carried out in a Boyden chambers as described before (Cho et al., 2012) with some modifications. Briefly, cell culture inserts (5 μm pore size) were used to form dual compartments (chambers) in a 24-well culture plate (Corning-Costar, Lowell, MA, United States). Macrophages prepared as described above (10^6^ cells in 0.2 ml of DMEM/F12–5% FBS) were loaded in the upper chamber and their migration was initiated with IEC supernatant prepared as above or with FMLP (10 μM) as a positive control, added to the bottom chamber. Cell migration was carried out for 24 h at 37°C and 5% CO_2_. The migrated macrophages were collected from the bottom chambers and counted with a hemocytometer, as detailed in previous studies (Vereyken et al., 2011; Unver et al., 2015). Basal macrophage migration observed in the absence of IEC supernatant was <20% of that induced with IEC supernatant and was subtracted from the data presented in the figures.

### Statistical Analysis

Results are expressed as mean ± SEM. The statistical differences between mean values were assessed by two-way ANOVA followed by Bonferroni test using graph-prism software. All results were considered statistically significant at *p* < 0.05 (one symbol), *p* < 0.01 (two symbols), or *p* < 0.001 (three symbols). The symbol (^∗^) was used to compare responses from *P2ry6*^-/-^ IEC with those from WT IEC. The symbol (#) was used to compare responses from stimulated IEC with those from non-stimulated IEC. The symbol (¶) was used to compare responses from poly(I:C)-treated IEC in the presence of inhibitors or antagonist with those from IEC treated with poly(I:C) alone.

## Results

### P2Y_6_ Receptor Is the Major Nucleotide Receptor Expressed in IEC

**FIGURE 1:**
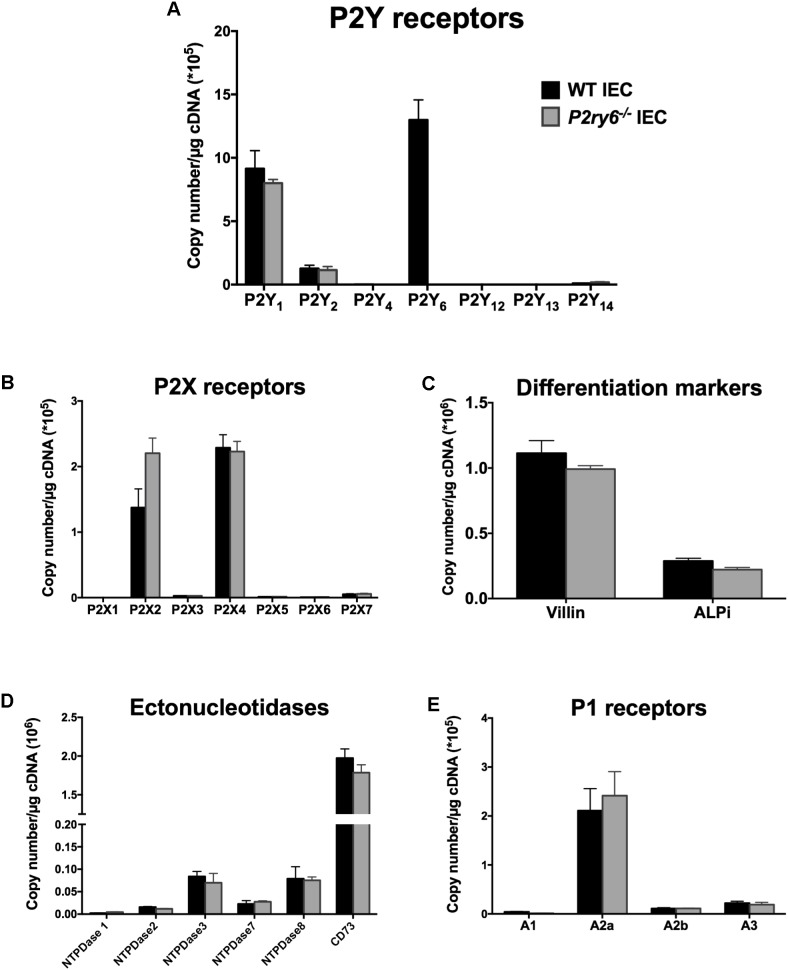
Characterization of primary IEC cultures. IECs were prepared and cultured as mentioned in the section “Materials and Methods” to obtain a monolayer of differentiated cells. RNA was isolated and the expression of P2Y receptors **(A)**, P2X receptors **(B)**, the differentiated epithelial cell markers villin and ALPi **(C)**, ectonucleotidases **(D)**, and P1 receptors **(E)** were analyzed by qRT-PCR. Data are normalized to GAPDH mRNA level. Data presented are the mean ± SEM of three independent experiments each with cells pooled from three mice.

We first evaluated the expression of P2 receptors in primary IECs isolated from WT colon. P2Y_6_ was the nucleotide receptor with the highest mRNA expression in these cells (13 × 10^5^ ± 1.7 × 10^5^ copies/μg of cDNA) followed by P2Y_1_ (9.5 × 10^5^ ± 1.4 × 10^5^ copies/μg of cDNA). There were also lower gene expressions of P2Y_2_ (1.2 × 10^5^ ± 0.1 × 10^5^ copies/μg of cDNA), P2X2 (1.37 × 10^5^ ± 0.22 × 10^5^ copies/μg cDNA), P2X4 (2.2 × 10^5^ ± 0.11 × 10^5^ copies/μg cDNA), and A_2A_ (2.1 × 10^5^ ± 0.5 × 10^5^ copies/μg cDNA) compared to the gene expression of P2Y_6_ receptor (**Figures 1A,B,E**).

### Characterization of *P2ry6^-/-^* IEC

Our main hypothesis is that P2Y_6_ receptors regulate functions of IEC related to inflammation. To address this, we used IEC isolated from mice deficient for the expression of P2Y_6_ receptor (*P2ry6*^-/-^). Microscopic observation of IEC and trypan blue staining did not show any apparent differences in the growth and differentiation of WT and *P2ry6*^-/-^ IEC (data not shown). In addition, IEC of both genotypes exhibited similar levels of mRNA of the IEC differentiation markers villin and intestinal alkaline phosphatase (ALPi) before and after differentiation. Before differentiation, the expression of villin and ALPi genes was below 0.04 × 10^6^ ± 0.002 × 10^6^ and 0.02 × 10^6^ ± 0.003 × 10^6^ copies/μg of cDNA, respectively (data not shown), for both WT and *P2ry6*^-/-^ IEC. After differentiation, the expression of these markers was as expected dramatically increased, over 1 × 10^6^ ± 0.02 × 10^6^ and 0.28 × 10^6^ ± 0.01 × 10^6^ copies/μg of cDNA for villin and ALPi, respectively, for both genotypes as measured by qRT-PCR (**Figure 1C**).

We then compared the expression of the receptors and enzymes involved in nucleotide signaling in WT and *P2ry6*^-/-^ IEC primary cultures. Aside P2Y_6_, we noted similar expression of all P2Y and P2X receptors at the gene level in IEC from both genotypes (**Figures 1A,B**) as well as of the plasma membrane-bound NTPDases and ecto-5’-nucleotidase (CD73) (**Figure 1D**). It is noteworthy that these ectonucleotidases not only hydrolyse the agonists of P2 receptors but also generate adenosine which activates P1 receptors. The analysis of P1 receptor expression reveals an important gene expression of the adenosine A_2A_ receptor (2.1 × 10^5^ ± 0.4 × 10^5^ for WT IEC and 2.5 × 10^5^ ± 0.4 × 10^5^ for *P2ry6*^-/-^ IEC) and nearly no expression of the other P1 receptors, in both WT and *P2ry6*^-/-^ IEC, which was in the order of 2 × 10^5^ copies/μg of cDNA (**Figure 1E**). Aside P2Y_6_, the minor variations in the expression of the genes tested in **Figure 1** between WT and *P2ry6*^-/-^ IEC were all not significantly different.

Together, these data show that *P2ry6*^-/-^ IECs are similar to WT IEC in all aspects analyzed, including gene expression of enzymes and receptors involved in nucleotide signaling, except, obviously, for the gene expression of P2Y_6_. We therefore used these cells to investigate the role of P2Y_6_ receptor in functions of IEC related to immune responses. More specifically, we investigated the implication of P2Y_6_ on IEC stimulated with TLR agonists to explore the contribution of this receptor in responses to PAMP.

### CXCL10 Expression and Secretion Are Augmented in *P2ry6^-/-^* IEC Stimulated by Poly(I:C)

We previously observed that the activation of P2Y_6_ was necessary to trigger the secretion of CXCL8 from human monocytes stimulated with TLR-2 ligand (Ben Yebdri et al., 2009). Moreover, P2Y_6_ activation increased CXCL8 secretion in the colonic tumor cell line Caco-2/15 (Grbic et al., 2008). Therefore, we hypothesized that in the absence of P2Y_6_ in IEC, chemokine secretion would be decreased upon TLR stimulation.

Intestinal epithelial cells express several TLRs at varying levels (Graves et al., 2014). The most important TLR receptors expressed in IEC lines, and which can affect the immune response, are TLR3, TLR4, and TLR5 (Bambou et al., 2004; Graves et al., 2014). We first investigated whether these TLRs induce chemokine expression in primary murine WT IEC and in IEC deficient for the expression of P2Y_6_. IECs were stimulated with the TLR agonists poly(I:C) (to activate TLR3), LPS (TLR4), and FLA-ST (TLR5). The expression of the chemokines KC, MIP-2, MCP-1, and CXCL10, known to be expressed by IEC in inflammatory conditions (Yang et al., 1997; Dwinell et al., 2001), was assessed by qRT-PCR.

**FIGURE 2:**
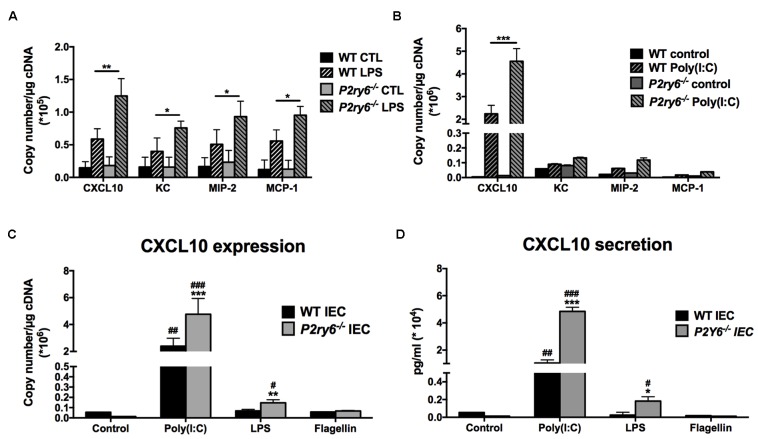
CXCL10 expression and secretion are increased in primary *P2ry6*^-/-^ IEC stimulated by a TLR3 ligand. **(A,B)** Primary IEC from WT or *P2ry6*^-/-^ mice was stimulated with LPS (0.1 μg/ml) **(A)** or poly(I:C) (10 μg/ml) **(B)** for 5 h then the expression of CXCL10, KC, MIP-2, and MCP-1 was analyzed by qRT-PCR. **(C,D)** Quantification of CXCL10 expression by qRT-PCR (**C**) and secretion by ELISA (**D**) in WT and *P2ry6*^-/-^ IEC stimulated with poly(I:C) (10 μg/ml), LPS (0.1 μg/ml), or flagellin (0.1 μg/ml) for 5 (qRT-PCR) or 24 h (ELISA), respectively. Data were normalized to GAPDH mRNA levels. Data presented are the mean ± SEM of five independent experiments for qRT-PCR and three independent experiments for ELISA, each with cells pooled from three mice. ^∗∗∗^*p* < 0.001 compared to WT IEC. ^##^*p* < 0.01, ^###^*p* < 0.01, poly(I:C) stimulated IEC compared to non-stimulated IEC.

The stimulation of primary IEC with LPS increased the expression of the four tested chemokines in both WT and *P2ry6*^-/-^ cells. The expression of KC, MIP-2, MCP-1, and CXCL10 was significantly increased in *P2ry6*^-/-^ IEC (0.75 × 10^5^± 0.05 × 10^5^, 0.93 × 10^5^± 0.2 × 10^5^, 0.95 × 10^5^± 0.1 × 10^5^, and 1.3 × 10^5^± 0.2 × 10^5^ copies/μg of cDNA, respectively) when compared to WT IEC treated with LPS (0.39 × 10^5^± 0.15 × 10^5^, *p* < 0.05; 0.5 × 10^5^± 0.2 × 10^5^, *p* < 0.05; 0.55 × 10^5^± 0.18 × 10^5^, *p* < 0.05, and 0.58 × 10^5^± 0.1 × 10^5^, *p* < 0.01, copies per μg of cDNA). CXCL10 was the highest chemokine expressed in both WT and *P2ry6*^-/-^ IEC (**Figure 2A**). The TLR3 agonist poly(I:C) also induced the expression of CXCL10 but more strongly [40-fold more than with LPS: 45 × 10^5^ and 1.3 × 10^5^ copies/μg of cDNA for poly(I:C) and LPS stimulation, respectively) (**Figures 2A,B**). As for LPS, poly(I:C) induced an increased expression and secretion of CXCL10 more prominently in *P2ry6*^-/-^ IEC than in WT IEC. The gene expression of CXCL10 was 4.5 × 10^6^± 0.5 × 10^6^ and 2.4 × 10^6^± 0.4 × 10^6^, *p <* 0.001, copies/μg of cDNA in *P2ry6*^-/-^ IEC and WT IEC, respectively. These data correlated with CXCL10 secretion which were 5.3 × 10^4^± 0.3 × 10^4^ and 1.8 × 10^4^± 0.2 × 10^4^ pg/ml, *p* < 0.001, in the supernatant of *P2ry6*^-/-^ IEC and WT IEC, respectively (**Figures 2B–D**). Stimulation with the purified flagellin FLA-ST showed low mRNA expression of the same chemokines (KC, MIP-2, and MCP-1) in both WT and *P2ry6*^-/-^ IEC (data not shown). We noted also low mRNA expression and protein secretion of CXCL10 in comparison with LPS and poly(I:C). The gene expression of CXCL10 was 0.08 × 10^6^± 0.001 × 10^6^ copies/μg of cDNA in both *P2ry6*^-/-^ IEC and WT IEC while for CXCL10 secretion was 250 ± 10 pg/ml in the supernatant of both *P2ry6*^-/-^ IEC and WT IEC (**Figures 2C,D**). These data show that murine primary IEC stimulated with the TLR-3 agonist poly(I:C) expressed high level of CXCL10 and that this production was more pronounced in the absence of P2Y_6_ receptor.

### Extracellular Nucleotides Are Involved in Poly(I:C)-Induced CXCL10 Release by IEC

In previous studies, we observed that the expression and secretion of the chemokine IL-8 induced by TLR2 and TLR4 activation required the concomitant activation of the nucleotide receptors P2Y_2_ and P2Y_6_ in human monocytes (Ben Yebdri et al., 2009). Therefore, we were not expecting that the expression of the chemokines tested here would be increased in *P2ry6*^-/-^ IEC as we observed for the four chemokines tested (**Figure 2**).

**FIGURE 3:**
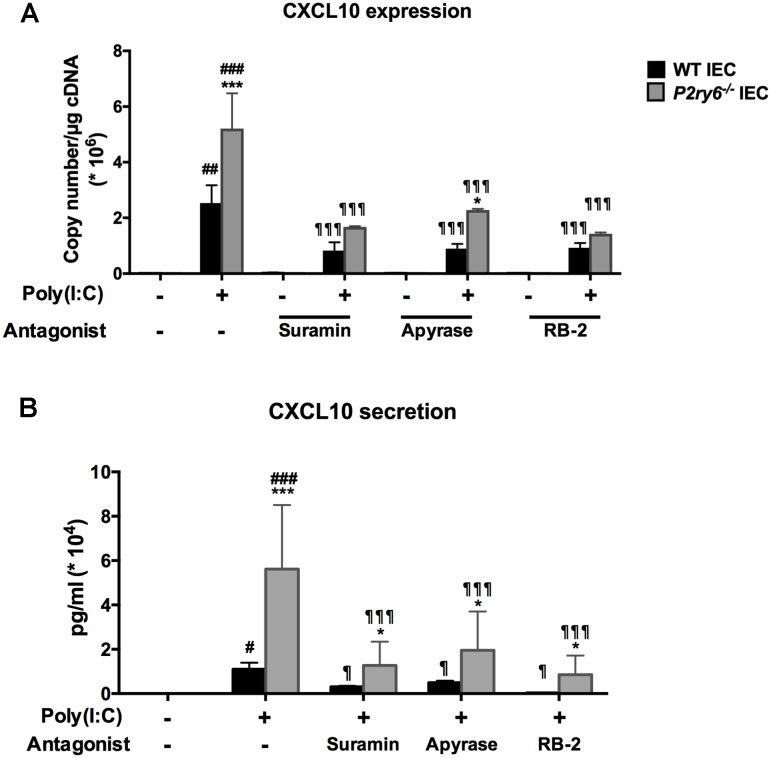
The TLR3 ligand poly(I:C) induces CXCL10 expression and secretion from IEC in a nucleotide-dependent manner. IECs were stimulated with poly(I:C) (10 μg/ml) for 5 h for qRT-PCR, or 24 h for ELISA, in the presence of the P2 blockers suramin (0.1 mM), RB-2 (0.1 mM), or apyrase (2 U/ml) added 20 min before stimulation. The CXCL10 expression **(A)** and secretion **(B)** were measured by qRT-PCR and ELISA, respectively. For qRT-PCR, data were normalized to actin mRNA levels. Data presented are the mean ± SEM of three independent experiments, each with cells pooled from three mice. One symbol *p* < 0.05, two symbols *p* < 0.01, and three symbols *p* < 0.001. ^∗^, stimulated IEC from *P2ry6*^-/-^ mice compared with WT; #, poly(I:C) stimulated IEC compared with non-stimulated IEC; and ¶, treated IEC in presence of inhibitors compared with poly(I:C) alone.

We then investigated whether extracellular nucleotides and their receptors played a role in TLR3-induced CXCL10 expression and secretion in both WT and *P2ry6*^-/-^ IEC. As seen in **Figure 3A**, IEC stimulated with poly(I:C), in the presence of the nucleotide scavenger apyrase or of the general P2 receptor antagonists suramin or RB-2, expressed much less CXCL10 mRNA level in both WT and *P2ry6*^-/-^ IEC. For WT IEC, CXCL10 gene expression was reduced from 2.4 × 10^6^± 0.7 × 10^6^ to 0.81 × 10^6^± 0.29 × 10^6^, *p* < 0.001; to 0.88 × 10^6^± 0.14 × 10^6^, *p* < 0.001 or to 0.9 × 10^6^± 0.15 × 10^6^, *p* < 0.001, copies/μg of cDNA in the presence of apyrase, suramin, or RB-2, respectively. For *P2ry6*^-/-^ IEC, CXCL10 gene expression was reduced from 5.0 × 10^6^± 1.3 × 10^6^ to 1.62 × 10^6^± 0.002 × 10^6^, *p* < 0.001; to 2.22 × 10^6^± 0.001 × 10^6^, *p* < 0.001 or to 1.33 × 10^6^± 0.005 × 10^6^, *p* < 0.001, copies/μg of cDNA in the presence of apyrase, suramin, or RB-2, respectively. Similar data were obtained at the protein level (**Figure 3B**). These data suggest that nucleotides participate in the regulation of TLR3-induced CXCL10 expression and secretion in IEC.

### Supernatants from Poly(I:C)-Stimulated IEC Induce Macrophage Migration *in Vitro*

**FIGURE 4:**
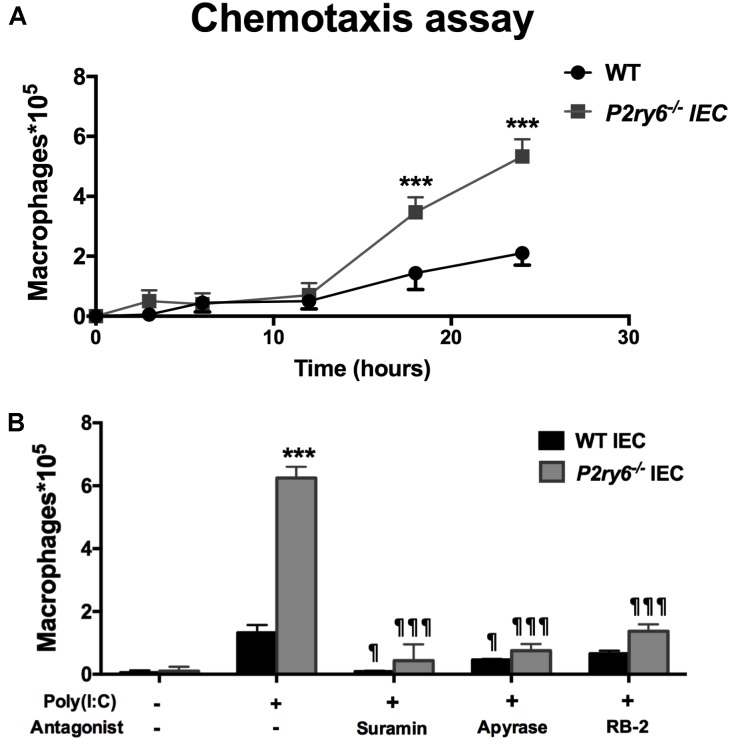
Supernatants from poly(I:C) stimulated IEC induce macrophage migration in a nucleotide-dependant manner. **(A)** Time course of bone marrow macrophage chemotaxis in Boyden chambers. The supernatant from poly(I:C)-treated WT or *P2ry6*^-/-^ *IEC* was added to the bottom chamber and 10^6^ BMDMs were applied to the upper chamber and allowed to migrate for 24 h. The migrated macrophages to the bottom chamber were counted with a hemocytometer and the measurements were reported on the *Y*-axis. Data presented are the mean ± SEM of three independent experiments, each with cells pooled from two mice. **(B)** Different IEC monolayers were preincubated for 20 min with the indicated P2 blockers or apyrase, at the same concentration as in **Figure 3**, prior stimulation with poly(I:C) for 24 h. These supernatants were added to the bottom chamber and macrophage chemotaxis was carried out for 24 h. Data presented are the mean ± SEM of four independent experiments, each with cells pooled from two mice. One symbol *p* < 0.05; three symbols *p* < 0.001. ^∗^, stimulated IEC from *P2ry6*^-/-^ mice compared with WT and ¶, treated IEC in the presence of inhibitors compared with poly(I:C) alone.

CXCL10 directs the chemotaxis of lymphocytes and macrophages. This can be observed *in vivo* when these cells migrate to an inflammatory site (Haemmerle et al., 2013; Zhao et al., 2017). We then questioned whether the supernatant of poly(I:C)-stimulated IEC, which contained significant amount of CXCL10 (**Figure 3B**), induced macrophage migration. Macrophage migration was assessed with a modified Boyden chamber system. The kinetics of cell migration presented in **Figure 4A** show that the supernatants of *P2ry6*^-/-^ IEC stimulated with poly(I:C) recruited significantly more macrophages (5.8 × 10^5^± 0.3 × 10^5^) than did the supernatants of poly(I:C)-stimulated WT IEC (2.1 × 10^5^± 0.1 × 10^5^) (60 vs. 20% of the macrophages added to the upper chamber had migrated to the lower chamber, respectively). This observation is in agreement with the higher concentration of CXCL10 measured in these supernatants. Note that we cannot exclude that other components and chemokines present in supernatants could also be implicated in this macrophage chemotaxis.

Then, we tested whether the inhibition of nucleotide signaling in poly(I:C)-treated IEC by apyrase or general P2 antagonists would diminish the ability of these cell supernatants to attract macrophages to the lower chamber. As shown in **Figure 4B**, the supernatants of both WT and *P2ry6*^-/-^ IEC stimulated with poly(I:C) in the presence of apyrase, suramin, or RB-2 recruited significantly fewer macrophages than did the supernatants of the cells stimulated in the absence of P2 blockers. Macrophage migration was reduced by about 94, 86, and 75% with supernatants from either WT or *P2ry6*^-/-^ IEC stimulated with poly(I:C) in the presence of suramin, apyrase, or RB-2, respectively. For example, the supernatant of *P2ry6*^-/-^ IEC stimulated with poly(I:C) alone induced the migration of 6.28 × 10^5^± 0.28 × 10^5^ macrophages which was reduced to 0.42 × 10^5^± 0.57 × 10^5^, 0.85 × 10^5^± 0.14 × 10^5^, or 1.42 × 10^5^± 0.14 × 10^5^ macrophages when the IECs were stimulated in the presence of suramin, apyrase, or RB-2, respectively. The *p*-values corresponding to these data are significant and are presented in **Figure 4B**.

These data show that supernatant of IEC stimulated with poly(I:C) induced macrophage migration (**Figure 4**) which correlated with the amount of CXCL10 detected in the supernatant of poly(I:C)-stimulated IEC in the presence/absence of P2 blockers (**Figure 3B**).

### Several P2 Receptors Are Involved in CXCL10 Expression and Secretion Induced by Poly(I:C) in *P2ry6^-/-^* IEC

**FIGURE 5:**
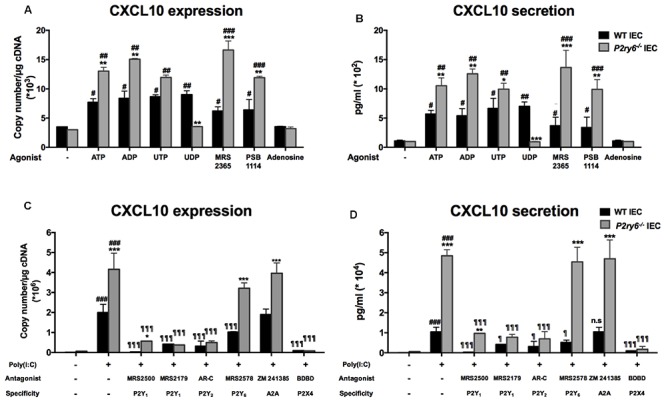
P2Y_6_ modulates nucleotide signaling-dependent CXCL10 expression and secretion from IEC. **(A,B)** IECs were stimulated either with nucleotides (ATP, ADP, UTP, or UDP) or adenosine (100 μM), or with specific agonist of P2Y_1_ (MRS 2365; 1 μM) or P2Y_2_ (PSB 114; 0.5 μM) receptors. Five or 24 h later the expression **(A)** or the secretion **(B)** of CXCL10 was evaluated by qRT-PCR and ELISA, respectively. **(C,D)** IECs were treated with poly(I:C) in the absence or presence of P receptor antagonists then CXCL10 expression **(C)** and secretion **(D)** were evaluated by qRT-PCR (5 h after the beginning of stimulation) and ELISA (24 h later), respectively. The antagonists used were MRS 2500 (5 μM) or MRS 2179 (0.15 μM) for P2Y_1_, AR-C 118925XX (2 μM) for P2Y_2_, MRS 2578 (1 μM) for P2Y_6_, or BDBD (100 μM) for P2X4, and Zm 241385 (10 nM) for A_2A_ receptor. Data are normalized to actin mRNA levels. Data presented are the mean ± SEM of five independent experiments for qRT-PCR and of three independent experiments for ELISA, each with cells pooled from three mice. One symbol *p* < 0.05; two symbols *p* < 0.01; three symbols *p* < 0.001. ^∗^, stimulated IEC from *P2ry6*^-/-^ mice compared with WT; #, poly(I:C) stimulated IEC compared with non-stimulated IEC; ¶, treated IEC in the presence of antagonists compared with poly(I:C) alone.

We then tested the involvement of different P2 receptors in the responses described above in both *P2ry6*^-/-^ and WT IEC. We first stimulated these cells with P1 and P2 receptor agonists. The natural ligand ATP, ADP, and UTP increased mRNA expression of CXCL10 in IEC from both, WT and *P2ry6*^-/-^ mice. Again the increase of expression was more prominent in *P2ry6*^-/-^ IEC (**Figure 5A**). The mRNA expression of CXCL10 was 13.0 × 10^3^± 0.4 × 10^3^ and 7.35 × 10^3^± 0.58 × 10^3^, *p* < 0.01, copies/μg of cDNA in *P2ry6*^-/-^ and WT IEC, respectively, when stimulated with ATP. When IECs were stimulated with ADP, it was 14.7 × 10^3^± 0.3 × 10^3^ and 8.2 × 10^3^± 1.2 × 10^3^, *p* < 0.01, copies/μg of cDNA for *P2ry6*^-/-^ and WT IEC, respectively. In addition, the specific agonist of P2Y_1_ (MRS 2365) and of P2Y_2_ (PSB1114) induced CXCL10 mRNA expression in *P2ry6*^-/-^ IEC more prominently than in WT IEC (16.2 × 10^3^± 1.5 × 10^3^ vs. 5.9 × 10^3^± 0.9 × 10^3^, *p* < 0.001, copies/μg of cDNA in the presence of MRS 2365 and 11.8 × 10^3^± 0.3 × 10^3^ vs. 6.2 × 10^3^± 1.6 × 10^3^, *p* < 0.01, copies/μg of cDNA in the presence of PSB1114. UDP, the specific agonist of P2Y_6_, also induced CXCL10 expression in WT IEC (8.5 × 10^3^± 1.1 × 10^3^ copies/μg of cDNA) compared to control IEC (3.6 × 10^3^± 0.1 × 10^3^ copies/μg of cDNA) (**Figure 5A**), which suggest that P2Y_6_ receptor plays a proinflammatory role in WT IEC. The *p*-value of UDP stimulation is significant and is presented in **Figure 5A**.

As expected, UDP did not induce any response in *P2ry6*^-/-^ IEC confirming the specificity of the assay (**Figure 5A**). In contrast, adenosine failed to induce a response in IEC of both WT and *P2ry6*^-/-^ mice (**Figure 5A**). Finally, **Figure 5B** shows that the CXCL10 protein level was in agreement with the level of expression detected in **Figure 5A**.

The candidate P2Y receptors expressed on IEC P2Y_1_, P2Y_2_, and P2Y_6_ as well as P2X4 and A_2A_ were tested with specific antagonists. MRS 2500 and MRS 2179 were used to block P2Y_1_, AR-C 118925XX to block P2Y_2_, MRS 2578 for P2Y_6_ receptor, 5-BDBD to prevent P2X4 activation, and Zm 241385 to block A_2A_ receptor. With the exception of the A_2A_ antagonist, all other selective and specific antagonists tested diminished CXCL10 mRNA expression in IEC upon stimulation with poly(I:C) (**Figure 5C**). For example, for *P2ry6*^-/-^ IEC, CXCL10 mRNA expression decreased from 4.3 × 10^6^± 0.8 × 10^6^ copies/μg of cDNA in IEC stimulated only with poly(I:C) to 0.45 × 10^6^± 0.08 × 10^6^, *p* < 0.001; 0.54 × 10^6^± 0.05 × 10^6^, *p* < 0.001; or 0.18 × 10^6^± 0.01 × 10^6^, *p* < 0.001, copies/μg of cDNA in the presence of MRS 2179, AR-C, or BDBD, respectively. No significant differences were noted between the responses produced by WT and *P2ry6*^-/-^ IEC in the presence of these antagonists except for P2Y_1_ and P2Y_6_ antagonists (**Figure 5C**). MRS 2578 did not affect significantly CXCL10 expression in *P2ry6*^-/-^ IEC showing the specificity of the assay. The nucleotide receptor P2X2 was not tested due to the lack of specific antagonists commercially available. We therefore cannot exclude that this receptor might also have an effect in CXCL10 expression. The A_2A_ antagonist had no effect on CXCL10 expression in either WT or *P2ry6*^-/-^ IEC (**Figure 5C**), which is in agreement with the absence of effect of adenosine on CXCL10 expression (**Figure 5A**). Similar data were obtained for all these antagonists at the protein level (**Figure 5D**).

These data suggest that several nucleotide receptors are involved in poly(I:C) stimulation and that these receptors are more stimulated in the absence of P2Y_6_ receptor in *P2ry6*^-/-^ *IEC*.

### FGF2 Signaling Pathway Is Upregulated in *P2ry6^-/-^* IEC

**FIGURE 6:**
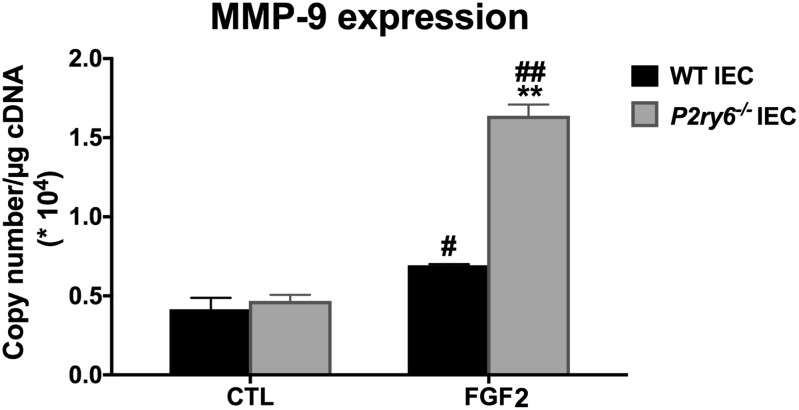
MMP9 gene expression induced by FGF2 is increased in *P2ry6*^-/-^ IEC. MMP-9 mRNA level in IEC stimulated with FGF2 (10 ng/ml) for 6 h was analyzed by qRT-PCR. Data were normalized to GAPDH mRNA levels. Data presented are the mean ± SEM of three independent experiments, each with cells pooled from three mice. One symbol *p* < 0.05; two symbols *p* < 0.01. ^∗^, stimulated IEC from *P2ry6*^-/-^ mice compared with WT; #, FGF2 stimulated IEC compared with non-stimulated IEC.

Given that there is no difference in the expression of P2 receptors and ectonucleotidases in WT and *P2ry6*^-/-^ *IEC*, we questioned whether the “primed” stimulation in *P2ry6*^-/-^ *IEC* was specific for nucleotide signaling or if it was also affecting other pathways not dependant on nucleotides. FGF2 stimulation for 6 h is known to induce the secretion of MMP-9 from mouse primary IECs (Song et al., 2015). While the stimulation of WT mouse IEC with FGF2 induced the expected gene expression of MMP-9 (0.67 × 10^4^± 0.02 × 10^4^, *p* < 0.05, copies/μg of cDNA), this response was significantly increased in *P2ry6*^-/-^ *IEC* (1.58 × 10^4^ ± 0.07 × 10^4^, *p* < 0.01, copies/μg of cDNA) (**Figure 6**).

## Discussion

In this study, we provide evidence that extracellular nucleotides regulate chemokine expression and secretion in primary IEC from mouse. We found that P2Y_6_ was the major nucleotide receptor expressed by primary IEC. We noted also the expression of P2Y_1_, P2Y_2_, P2X2, P2X4, and of the adenosine receptor A_2A_ in IEC. Previous studies also showed the presence of P2Y_6_ in Caco2/15 and IEC-6, which are human and rat intestinal cell lines, respectively (Grbic et al., 2008; Nakamura et al., 2013).

We next investigated the role of nucleotides in chemokine secretion by IEC and the implication of P2Y_6_ in this function. The activation of P2Y_6_ receptor with its specific agonist UDP led to a significant increase of CXCL10 expression and secretion in WT IEC. These data are in line with other studies which showed that P2Y_6_ had proinflammatory functions such as the induction of expression and secretion of proinflammatory cytokines and chemokines (Grbic et al., 2008; Ben Yebdri et al., 2009; Hao et al., 2014).

For the following studies, we purified primary IEC from *P2ry6*^-/-^ mice to verify whether these cells could still release CXCL10 in the absence of P2Y_6_. To mimic inflammatory conditions, we used TLR activation as stimuli as mounting evidences suggest that their activation is often, if not always, associated with the release of nucleotides from affected cells (Yin et al., 2007; Ben Yebdri et al., 2009; Kukulski et al., 2010; Ivison et al., 2011). This process is important as extracellular nucleotides, by activating P2 receptors, have been shown to be necessary for the optimal proinflammatory effects induced by TLR activation. In this study, we found that TLR3 and TLR4 stimulation led to an up-regulation of CXCL10 expression from IEC. The other chemokines tested, KC, MIP-2, and MCP-1, also followed this tendency with an expression level much lower than that of CXCL10. The group of Proost et al. (2003) has reported an increased expression of CXCL10 in fibroblasts and peripheral blood mononuclear cells upon stimulation by TLR3 ligand and IFN-gamma.

The TLR3 stimulation with poly(I:C) led to high CXCL10 secretion from IEC of both genotypes (WT and *P2ry6*^-/-^). The inhibition of this response by general P2 blockers suggests that nucleotides are implicated in these responses. We then further studied the implication of nucleotide signaling in this process. First, the activation of each P2 receptor expressed by IEC, but not P1 receptor, resulted in a significant increase of CXCL10 expression and secretion. CXCL10 expression and secretion induced by a TLR3 agonist were decreased in the presence of the general P2 receptor antagonists suramin and RB-2, and also by the nucleotide scavenger apyrase in both WT and *P2ry6*^-/-^ IEC (**Figure 3**). In all the experiments above, the level of CXCL10 measured correlated with the level of macrophage chemotaxis reported in **Figure 4**.

These data suggest that nucleotides play a key role in inducing chemokine expression and secretion by IEC under PAMP stimulation and that several P2 receptors are involved in this response. In agreement with these results, we and others have shown that extracellular nucleotides are implicated in chemokine expression and cell migration. In addition, Kukulski et al. (2010) showed that nucleotides and P2 receptors were required for IL-8 to induce neutrophil migration. In agreement with the above observations, nucleotide hydrolysis by ectoenzymes controlled IL-8 production in the colonic human cell line HT-29 (Bahrami et al., 2014). In addition to these observations, Ivison et al. (2011) demonstrated that ATP regulated the inflammatory response to flagellin via TLR5 activation in immortalized human IEC.

One surprising observation presented in this paper was that IEC that do not express P2Y_6_ induced greater levels of chemokines expression in response to TLR agonists, suggesting that the presence of P2Y_6_ negatively regulates the reactivity of other receptors at the surface of IEC. Indeed, we observed that in the absence of P2Y_6_, other P2 receptors expressed on IEC such as P2Y_1_ and P2Y_2_ induced a stronger response than what was measured in WT IEC that express P2Y_6_ normally. It is noteworthy to mention that genes of all nucleotide receptors were expressed at similar levels in both WT and *P2ry6*^-/-^ IEC excluding a compensatory mechanism involving a greater expression of another P2 receptor in *P2ry6*^-/-^ IEC. It is noteworthy that the pharmacological inhibition of P2Y_6_ activation in WT IEC did not reproduce the stimulatory effect of *p2ry6* gene deletion on the expression and secretion of CXCL10. The P2Y_6_ antagonist reduced the poly(I:C)-stimulated expression and secretion of CXCL10. Pharmacological inhibition and gene targeting differ in that, while the MRS 2578 effect is punctual, that of gene deletion is permanent. It is possible that despite the fact that *P2ry6*^-/-^ and WT IEC are equally differentiated, *P2ry6*^-/-^ IEC respond more vigorously to stimulation because P2Y_6_ receptors regulate the expression of the component of TLR3 signaling pathway. The examination of this hypothesis is beyond the scope of the present study and will be investigated subsequently.

As the activation of several P2 receptors seemed to be regulated by the presence of P2Y_6_, we questioned whether this effect also affected pathways independent of nucleotide signaling. Indeed, FGF2 stimulation induced a greater gene expression of MMP-9 in *P2ry6*^-/-^ IEC than in WT IEC (**Figure 6**). Altogether, these data suggest that the presence of P2Y_6_ regulates the activation of multiple receptors at the surface of IEC in a general manner which may affect the homeostasis of the cells.

Another explanation could be that P2Y_6_ triggers not only a signaling pathway which controlled positively chemokine production but also a negative loop to prevent overstimulation of the cells. The interaction between intracellular signaling of P2X and P2Y receptors has already been observed in several cell types (Erb and Weisman, 2012). For example, crosstalk between P2 receptors has previously been reported by Bernier et al. (2013) who showed that P2X4 activation is controlled by P2Y_6_ in microglia possibly through depletion of membrane phosphoinositide resulting from phospholipase C activation by P2Y_6_. In a human osteoblast cell line (Jorgensen et al., 1997), Ihara et al. (2005) observed that the activation of P2X4, P2X5, and P2X6 with ATP resulted in IL-6 secretion and that this response was inhibited by the antagonist of P2Y receptors. Activation of human P2Y_1_ and P2Y_2_ receptors has been shown to potentiate subsequent function-mediated Ca^2+^ signaling which is related to P2X function (Bowler et al., 1999).

As mentioned above, we also observed that CXCL10 expression was decreased in the presence of P2X4, P2Y_1_, and P2Y_2_ antagonists in both WT and *P2ry6*^-/-^ IEC. The blockade of an important part of the response by an antagonist of a single-nucleotide receptor may suggest a complex function of nucleotides in cytokine expression in IEC. The fact that these receptors are coupled to different pathways may partly explain this observation. P2Y_1_ is coupled to G_q_ protein, P2Y_2_ is coupled to G_0_, P2Y_6_ to Gq/_11_ (Okada et al., 2002; Ando et al., 2010; Harden et al., 2010; Ishida et al., 2013) while P2X4 is a ligand-gated ion channel (North, 2002). The co-stimulation of different nucleotide receptors to get a function has been well documented in platelets which express P2Y_1_, P2Y_12_, and P2X1 receptors (Daniel et al., 1998; Jin et al., 1998). The co-stimulation of P2Y_1_ and P2Y_12_ by the same agonist ADP is necessary to induce platelet activation. While P2Y_1_ is coupled to phospholipase C, P2Y_12_ is linked with the inhibition of adenylyl cyclase. If one of these two pathways is blocked, there will be no platelet activation (Jin and Kunapuli, 1998).

It is noteworthy that a cross talk between P2Y receptors and P2X receptor ion channels has also been noted in *Xenopus* oocytes. In these cells, P2X1 receptor activated by ATP led to a transient inward current that is rapidly desensitized by ATP itself (Rettinger and Schmalzing, 2003). Interestingly, the co-expression and co-activation of either P2Y_1_ or P2Y_2_ inhibit P2X1 receptor desensitization. The mechanism of P2Y receptor-mediated inhibition of P2X1 receptor desensitization does not appear to involve direct phosphorylation of the P2X1 receptor but does involve protein kinase activity, perhaps mediated by an accessory protein (Jones et al., 2014). Although these mechanisms cannot be shared completely with the data presented here in IEC, it still shows that nucleotide signaling is complex and that several nucleotide receptors may often be needed to control specific effects.

## Conclusion

The data presented in this study support the view that nucleotide signaling can contribute to leukocyte recruitment to the intestinal epithelium via CXCL10 secretion by IEC. This mechanism involves P2Y_6_. The results presented here also show that P2Y_1_, P2Y_2_, and P2X4 also regulate CXCL10 secretion in these cells, especially in the absence of P2Y_6_. Indeed, in *P2ry6*^-/-^ IEC, activation of nucleotide receptors induced a stronger expression and secretion of the chemokine CXCL10 when compared to WT IEC. The *P2ry6*^-/-^ IEC also responded more vigorously to a non-nucleotide receptor as demonstrated with FGF2 that induced a stronger expression of MMP9 gene in the *P2ry6*^-/-^ IEC. Therefore, P2Y_6_ receptors may not only induce effects such as chemokine release but may also act as a regulator of IEC homeostasis by preventing these cells to over react to various stimuli.

## Author Contributions

MS conceived the proposal study design, performed all the experiments, analyzed the data, and wrote the first draft of the manuscript. AT helped MS to perform epithelial cell culture and qRT-PCR experiments. JP took care of mice reproduction. BR provided P2Y_6_-deficient mice and helped with the analysis of the data and with manuscript writing. JS supervised the study.

## Conflict of Interest Statement

The authors declare that the research was conducted in the absence of any commercial or financial relationships that could be construed as a potential conflict of interest.
